# PINK1 deficiency impairs osteoblast differentiation through aberrant mitochondrial homeostasis

**DOI:** 10.1186/s13287-021-02656-4

**Published:** 2021-11-25

**Authors:** So-Young Lee, Hyun-Ju An, Jin Man Kim, Min-Ji Sung, Do Kyung Kim, Hyung Kyung Kim, Jongbeom Oh, Hye Yun Jeong, Yu Ho Lee, Taeyoung Yang, Jun Han Kim, Ha Jeong Lim, Soonchul Lee

**Affiliations:** 1 Department of Internal Medicine, CHA Bundang Medical Center, CHA University School of Medicine, Seongnam-si, 13496 Republic of Korea; 2 Department of Orthopaedic Surgery, CHA Bundang Medical Center, CHA University School of Medicine, 59 Yatap-ro, Bundang-gu, Seongnam-si, 13496 South Korea; 3 Department of Oral Microbiology and Immunology, School of Dentistry, Seoul National University, Seoul, 03080 Republic of Korea; 4 CHA Graduate School of Medicine, 120 Hyeryong-ro, Pocheon, 11160 Republic of Korea; 5 Department of Pathology, Kyung Hee University Hospital at Gangdong, Kyung Hee University, College of Medicine, Seoul, 05278 Republic of Korea

**Keywords:** Mitochondria, Osteogenesis, Osteoporosis, PINK1

## Abstract

**Background:**

PTEN-induced kinase 1 (PINK1) is a serine/threonine-protein kinase in mitochondria that is critical for mitochondrial quality control. PINK1 triggers mitophagy, a selective autophagy of mitochondria, and is involved in mitochondrial regeneration. Although increments of mitochondrial biogenesis and activity are known to be crucial during differentiation, data regarding the specific role of PINK1 in osteogenic maturation and bone remodeling are limited.

**Methods:**

We adopted an ovariectomy model in female wildtype and *Pink1*^*−/−*^ mice. Ovariectomized mice were analyzed using micro-CT, H&E staining, Masson’s trichrome staining. RT-PCR, western blot, immunofluorescence, alkaline phosphatase, and alizarin red staining were performed to assess the expression of PINK1 and osteogenic markers in silencing of PINK1 MC3T3-E1 cells. Clinical relevance of PINK1 expression levels was determined via qRT-PCR analysis in normal and osteoporosis patients.

**Results:**

A significant decrease in bone mass and collagen deposition was observed in the femurs of *Pink1*^*−/−*^ mice after ovariectomy. Ex vivo, differentiation of osteoblasts was inhibited upon Pink1 downregulation, accompanied by impaired mitochondrial homeostasis, increased mitochondrial reactive oxygen species production, and defects in mitochondrial calcium handling. Furthermore, PINK1 expression was reduced in bones from patients with osteoporosis, which supports the practical role of PINK1 in human bone disease.

**Conclusions:**

In this study, we demonstrated that activation of PINK1 is a requisite in osteoblasts during differentiation, which is related to mitochondrial quality control and low reactive oxygen species production. Enhancing PINK1 activity might be a possible treatment target in bone diseases as it can promote a healthy pool of functional mitochondria in osteoblasts.

**Supplementary Information:**

The online version contains supplementary material available at 10.1186/s13287-021-02656-4.

## Introduction

Bones in our body constitute a part of the vertebrate skeleton and maintain their structure by constant bone remodeling throughout the life span [1]. Osteoblasts are responsible for synthesizing bone matrix and carrying out mineralization against the action of osteoclasts, which break down and resorb bones during bone remodeling [2]. In this context, osteoblast differentiation from progenitor cells is seminal for preventing loss of bone mass, which is observed in patients with osteoporosis [3].

Mitochondria are essential organelles for eukaryotic cells that are in charge of energy production, storage of calcium ions, and regulation of cell death [4]. When cellular differentiation occurs, many adenosine triphosphates (ATPs) supplied by mitochondria are needed to change cellular structure and alter function [5]. In a previous experimental study, the suppression of mitochondrial activity significantly limited osteoblast differentiation [6]. The mitochondrial oxidative phosphorylation system (OXPHOS) generates ATPs to fuel cells; reactive oxygen species (ROS) are generated as by-products of this process [6]. Notably, epidemiological studies in humans and recent mechanistic evidence in animals revealed that an increase in ROS influences bone cells and leads to defects in osteogenic formation [7].

PTEN-induced kinase 1 (PINK1) is a serine/threonine-protein kinase in mitochondria that is critical for mitochondrial quality control; PINK1 triggers mitophagy, a selective autophagy of mitochondria and is involved in mitochondrial regeneration at the same time [8–12]. Accumulating evidence suggests that PINK1-associated mitochondrial quality control is relevant to human diseases such as neurodegenerative [13–15], pulmonary [16], heart [17], and kidney [18] diseases. However, the specific role of PINK1 in osteoblasts and bone remodeling has not been described yet.

In the current study, we investigated bone mass and mitochondria in osteogenic cells of ovariectomized mice with genetic deletion of PINK1. We demonstrated that downregulation of PINK1 impaired mitochondrial homeostasis, increased mitochondrial ROS production, and inhibited osteoblast differentiation. Furthermore, we examined the level of PINK1 expression in patients with osteoporosis.

## Results

### Increased bone loss in ***Pink1***^−/−^ mice after ovariectomy

**Fig. 1:**
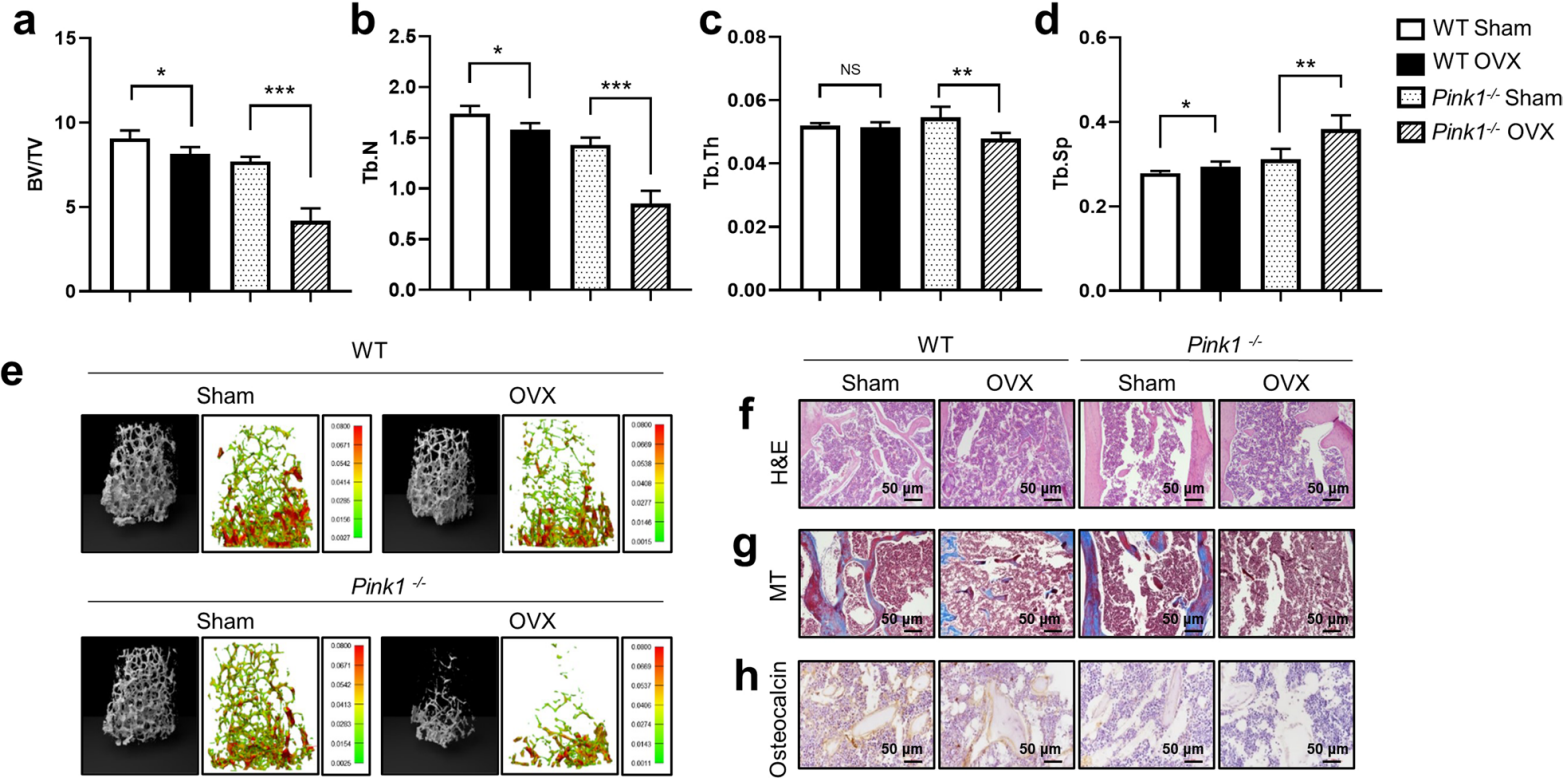
Increased bone loss in *Pink1*^−/−^ mice after ovariectomy (OVX). The following parameters were calculated during the analysis of trabecular bone and architecture: **a** bone volume per total volume (BV/TV), **b** trabecular number (Tb.N), **c** trabecular thickness (Tb.Th), and **d** trabecular spacing (Tb.Sp). **e** Representative images of μCT and finite element analysis of femoral trabecular bones. Representative photographs of **f** hematoxylin–eosin (H&E), **g** Masson’s trichrome (MT), and **h** immunohistochemical staining of osteocalcin in femurs from WT or *Pink1*^–/–^ mice after OVX or sham operation. Data have been expressed as mean ± SEM; *P < 0.05; **P < 0.01; ***P < 0.001; *n* = 6; scale bar = 50 μm

We first examined the effect of *Pink1* deficiency on ovariectomy (OVX)-induced bone loss. *Pink1-*knockout (KO) mice were generated and their genotype was identified using western blotting (Additional file 1: Fig. S1). *Pink1*^−/−^ mice developed normally without any gross abnormalities. We established osteoporosis models by OVX in Wild-type C57BL/6 mice (WT) and *Pink1*^−/−^ mice and analyzed their bone parameters at 4 weeks after surgery. Data from μCT imaging of mouse femurs showed a significant reduction in the trabecular bone volume fraction (BV/TV, Fig. 1a), number (Tb.N, Fig. 1b), and thickness (Tb.Th, Fig. 1c), along with a significant increase in trabecular separation (Tb.Sp, Fig. 1d) in the *Pink1*^−/−^ OVX group. On comparing among groups in terms of percentage changes, BV/TV (Fig. 1a, right panel) and Tb.N (Fig. 1b, right panel) were found to be lower in the *Pink1*^−/−^ mice group after OVX than in the *Pink1*^−/−^ mice. Consistently, the *Pink1*^−/−^ OVX group displayed a greater percentage change in Tb.Sp (Fig. 1d, right panel) than the WT OVX group.

Representative three-dimensional (3D) μCT images and hematoxylin and eosin (H&E)-stained trabecular bone sections from the femur revealed markedly osteopenic phenotypes due to thinner and fewer trabecular bones in the *Pink1*^−/−^ OVX group, as compared to the WT OVX group and sham-operated counterpart (Fig. 1e-f). Histological analysis of Masson’s trichrome (MT) staining revealed significantly decreased deposition of collagen fiber in the bones of the *Pink1*^−/−^ OVX group, which is produced by mature osteoblasts (Fig. 1g). Immunohistochemical staining showed lower expression of osteocalcin (Ocn), a hormone released by osteoblasts, in the femurs of *Pink1*^−/−^ mice (Fig. 1h). The number of osteoblasts displaying staining for Ocn also decreased significantly in the bones of Pink1^−/−^ mice (Additional file 1: Fig. S2). As illustrated by these results, PINK1 deficiency augments bone loss after OVX and is related to osteoblast abnormality.

### PINK1 regulates osteoblast differentiation

**Fig. 2:**
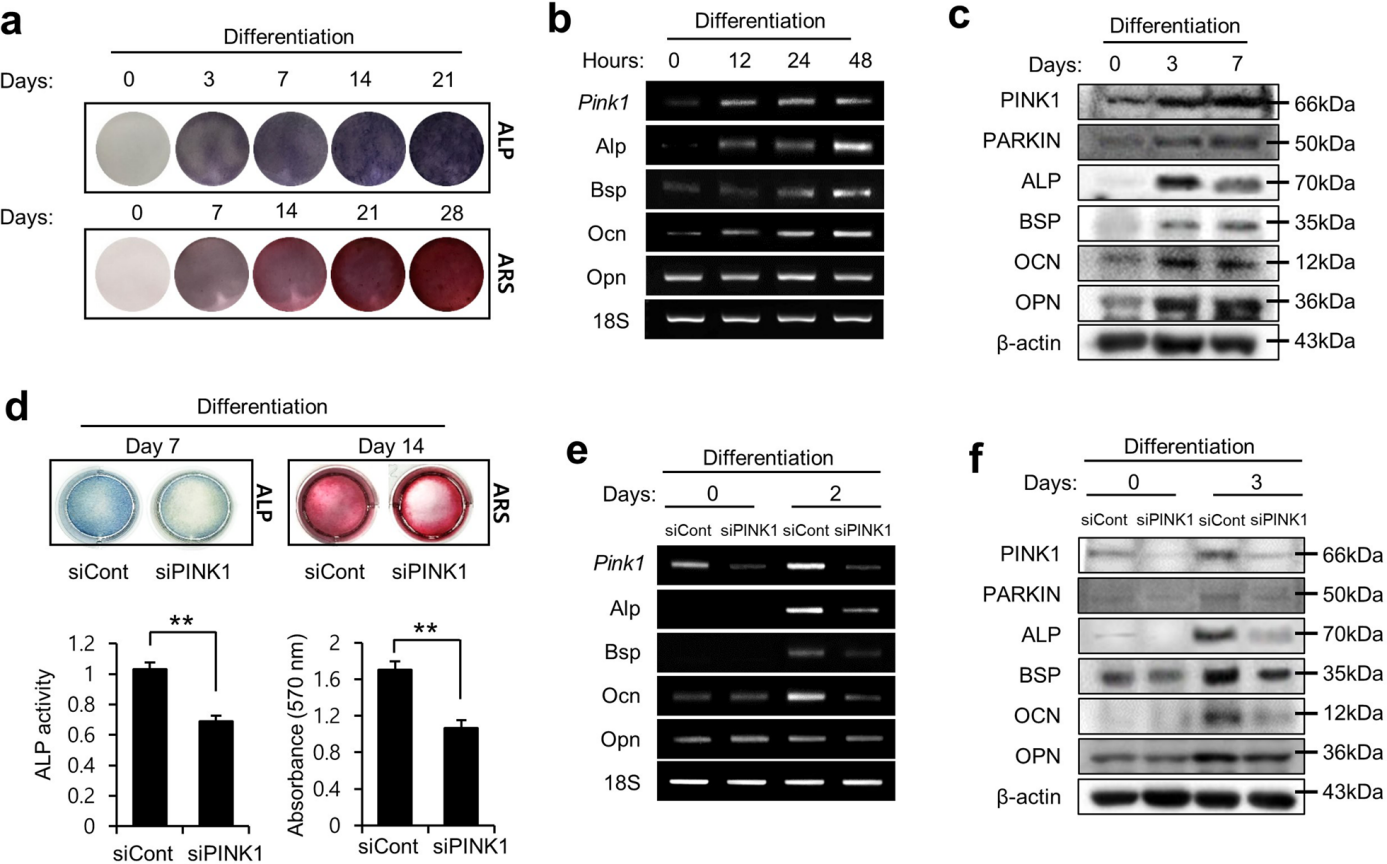
PINK1 is required for osteoblast differentiation. **a** MC3T3-E1 cells were induced to differentiate, followed by staining of the cells using alkaline phosphate (Alp) and Alizarin Red S (ARS). **b** mRNA levels of *Pink1* and other markers of mature osteoblasts in MC3T3-E1 cells after osteogenic induction. **c** Protein levels of PINK1 and other osteogenic markers were analyzed using western blotting after osteogenic induction. **d** Alp and ARS staining of MC3T3-E1 cells that were differentiated after treatment with *Pink1* siRNA. Histograms show ALP activity and quantification of Alizarin red S staining by spectrophotometry. **e** The effects of *Pink1* siRNA on the mRNA levels of marker genes of mature osteoblasts. **f** The effects of *Pink1* siRNA on protein levels of osteogenic markers in cells. Bsp, bone sialoprotein; Ocn, osteocalcin; Opn, osteopontin; Pink1, PTEN-induced kinase 1

To initiate osteogenic differentiation, MC3T3-E1 cells were supplemented with conditioned medium for the indicated periods. Alkaline phosphatase (Alp) and Alizarin Red S (ARS) staining indicated the ability of osteoblast precursor cells to differentiate and form a mineralizing matrix as mature functional osteoblasts (Fig. 2a). Furthermore, PINK1 expression at the mRNA and protein levels was found to be increased during osteoblastic differentiation along with the expression of osteogenic markers, including Alp, bone sialoprotein (Bsp), Ocn, and osteopontin (Opn) (Fig. 2b–c). Preosteoblastic MC3T3-E1 cells, transfected with either *Pink1* siRNA or negative control (NC)-siRNA, were grown in osteogenic differentiation media. After 7 and 14 days, Alp- and ARS-stained cells showed lower levels of mineralization in *Pink1*-depleted MC3T3-E1 cells than in the control cells. Repressed staining of these activities (Fig. 2d) indicates that PINK1 knockdown suppressed the osteoblastic differentiation of MC3T3-E1 cells. We then induced MC3T3-E1 cells to differentiate into an osteogenic lineage and detected the expression of osteogenic markers at both the mRNA and protein levels. After osteogenic induction, the expression levels of Alp, Bsp, Ocn, and Opn were found to be significantly lower in the *Pink1*-depleted cells than in the NC siRNA-transfected cells (Fig. 2e–f). Also, after osteogenic induction, the expression levels of Alp, Bsp, Ocn, and Opn were found to be significantly higher in the *Pink1*-overexpressed cells than in the empty vector transfected cells (Additional file 1: Fig. S3). Taken together, these results imply that endogenous PINK1 plays an important role as a positive regulator of osteogenic differentiation.

### Knockdown of PINK1 impairs mitochondrial homeostasis and aggravates ROS production during osteoblast differentiation

Mitochondria perform their roles during cell differentiation by maintaining their mass, accommodating dynamic changes in their shapes, and controlling ROS production [19]. Activation of PINK1 has been known to be critical in mitochondrial quality control [12]. We hypothesized that PINK1 deficiency induces mitochondrial abnormalities and results in ROS overproduction in osteogenic lineage cells.

**Fig. 3:**
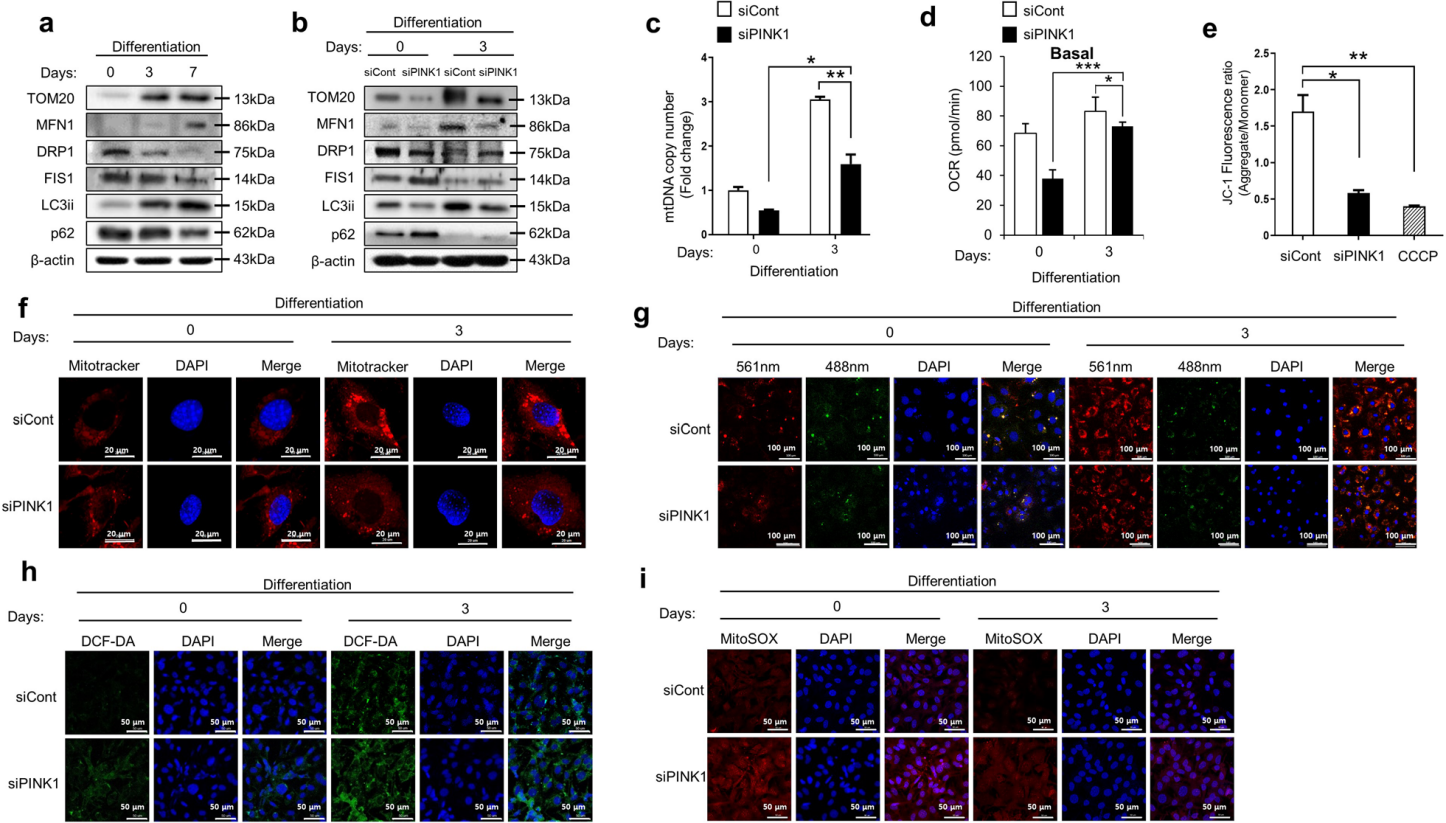
Effect of *Pink1* siRNA on mitochondrial homeostasis and ROS production during osteoblast differentiation. **a** Expression levels of mitochondria functional unit (Tom20), profusion (Mfn1), pro-fission (Drp1 and Fis1), and autophagy (LC3ii and p62) related proteins in MC3T3-E1 cells after osteogenic induction. **b** The effects of *Pink1* siRNA on protein levels of mitochondria functional unit, profusion, pro-fission, and autophagy-related proteins in cells after differentiation for 3 days. **c** mtDNA copy number in cells after treatment with *Pink1* siRNA. **d** The effects of *Pink1* siRNA *on* Mitochondrial respiration, reflected by the oxygen consumption rate (OCR) level in cells after differentiation for 3 days. The oxygen consumption rate (OCR) was analyzed using a Seahorse XF-24 analyzer. Rates of basal respiration were quantified by normalization of OCR levels to total protein levels obtained from O.D. values. **e** Mitochondrial membrane potential (ΔΨm) was studied by measuring JC-1 uptake in MC3T3-E1 cells after osteogenic induction with or without *Pink1* siRNA treatment. **f** Representative confocal images of MitoTracker™ (red) in MC3T3-E1 cells with or without treatment with *Pink1* siRNA. Scale bar = 20 μm. **g** Representative confocal images of mtKeima in MC3T3-E1 cells with or without treatment with *Pink1* siRNA. Scale bar = 20 μm. **h**–**i**) Representative confocal images of 2,7-dichlorodihydrofluorescein diacetate (H2-DCFDA; green) and MitoSOX™ (red), showing mitochondrial and intracellular ROS production in MC3T3-E1 cells with or without treatment with *Pink1* siRNA. mtDNA, mitochondrial DNA; CCCP, carbonyl cyanide m-chlorophenyl hydrazone. Data have been expressed as mean ± SEM; *P < 0.05; **P < 0.01; n = 4; scale bar = 50 μm

Figure 3a shows a gradual rise in the expression of Tom20, a subunit of mitochondrial import receptor, suggesting increased mitochondrial functional mass in the process of osteoblastic differentiation. During osteogenic maturation, mitochondrial pro-fission proteins Drp1 and Fis1 were downregulated, while the profusion protein, Mfn1 was upregulated, reflecting mitochondrial networking for high respiratory activity (Fig. 3a) [20]. Microtubule-associated protein light chain 3 (LC3) is a marker of autophagosomes and LC3ii is a lipid-modified form of LC3. The adaptor protein, p62, is degraded in cells with normal autophagy flux. Western blotting with anti-LC3ii and anti-p62 antibodies showed increased autophagic activity during osteogenic induction in preosteoblast cells (Fig. 3a).

However, when the expression of PINK1 was suppressed, the increase in mitochondrial functional mass was reduced compared to that in control cells during osteoblastic differentiation, as shown using western blotting and mitochondrial DNA (mtDNA) copy number assay (Fig. 3b–c). We analyzed mitochondrial activity in the PINK1-depleted cells using a Seahorse XF analyzer to determine the indicator of mitochondrial respiration. Knockdown of PINK1 was observed to have a significant decrease in mitochondrial respiration suggesting failure of increment of functional mitochondrial mass (Fig. 3d). MitoTracker™ Red staining confirmed that treatment with *Pink1* siRNA inhibited the increase in functional mitochondria during the osteogenic process (Fig. 3f). Knockdown of PINK1 increased the levels of pro-fission proteins, but decreased the profusion proteins and limited the autophagic activity, at 3 days after osteoblastic induction (Fig. 3b).

We performed the JC-1 assay to evaluate mitochondrial membrane potential (ΔΨm) as an indicator of mitochondrial viability and function [21]. Figure 3e shows that treating preosteoblastic cells with *Pink1* siRNA aggregated mitochondrial depolarization, similar to treatment with CCCP (carbonyl cyanide m-chlorophenyl hydrazine), a potent mitochondrial membrane disruptor. We next used the mitochondria-targeted Keima (mt-Keima) probe to detect functional mitophagy. We found that the occurrence of red mt-Keima punta, indicative of the presence of mitochondria in maturing autolysosomes, decreased in preosteoblastic cells after treatment with *Pink1* siRNA during osteogenic differentiation (Fig. 3g). Mitophagy is selective autophagy that removes defective mitochondria in cells [22]. Finally, as expected, downregulation of PINK1 induced robust intracellular ROS (DCF-DA, green) and mitochondrial superoxide accumulation (MitoSOX™, red) in cells during osteoblastic differentiation (Fig. 3h–i).

Taken together, lower expression of PINK1 is associated with lower functional mitochondrial mass, poorer networking, and insufficient selective removal of damaged mitochondria, which culminates in overproduction of mitochondrial ROS.

### PINK1 deficiency alters mitochondrial calcium handling dynamics

**Fig. 4:**
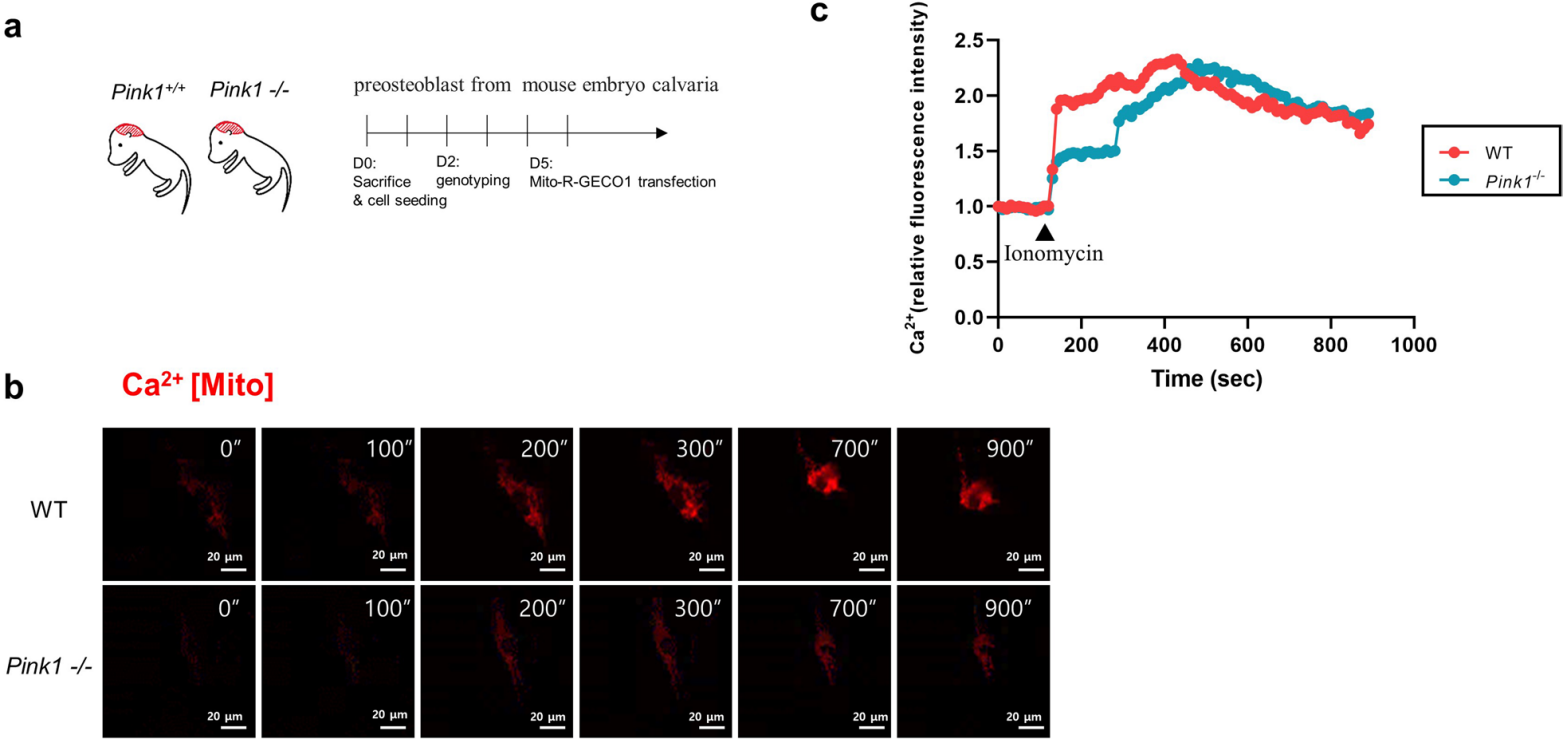
PINK1 deficiency impairs mitochondrial calcium uptake. **a** Experimental protocol for live cell imaging of preosteoblasts from mouse embryo calvaria. **b** Fluorescent time-lapse images of mito-LAR-GECO1.2-expressing preosteoblasts at the indicated times post-ionomycin treatment (500 nM). **c** Quantified intensity of Mito-R-GECO1 fluorescence in each indicated preosteoblast. Data from three representative cells have been shown for each treatment. Trajectories for each cell have been represented in a different color. Fluorescence intensity, quantified as average pixel intensity per cell, using the same confocal imaging system. Scale bar = 20 μm

Intracellular calcium handling is one of the important roles of mitochondria [23]. Mitochondria act as calcium buffers to strictly regulate cytoplasmic calcium concentration [24]. Healthy mitochondria with normal Δ*Ψm* are essential for appropriately transporting cytoplasmic calcium. To characterize the effects of PINK1 deficiency on the capacity of mitochondrial calcium handling, we monitored intra-mitochondrial calcium dynamics upon cytosolic calcium influx. To this end, ionomycin (ionophore) was treated with *Pink1*^*−/−*^ and WT preosteoblasts, and mitochondrial calcium levels were measured by expressing the mitochondria-targeted genetically encoded calcium indicator (mito-LAR-GECO1.2) [25] (Fig. 4a). After ionomycin treatment, there was an immediate increase in the mitochondrial calcium level in WT osteoblasts; the peak of the calcium level reached at ~ 300 s (Fig. 4b, c). Slower uptake dynamics of calcium level were detected in *Pink1*^*−/−*^ preosteoblasts, but the peak level and persistency were comparable to those of the wild-type cells (Fig. 4b, c). These data indicate impaired mitochondrial function in the calcium uptake of PINK1-deficient preosteoblasts.

### Increased abnormal mitochondria in osteoblastic cells of PINK1-knockout mice

Mitochondria form large tubular assemblies and have a highly flexible and dynamic network architecture which changes in response to its energy demands or to resist unfavorable environments [4]. Mitochondrial fusion contributes to the maintenance of bioactivity of the mitochondrial network and is critical for cell survival and growth [26, 27]. On the other hand, fragmented mitochondria are associated with mitochondrial outer membrane permeabilization, ROS production, and cell death [28].

**Fig. 5:**
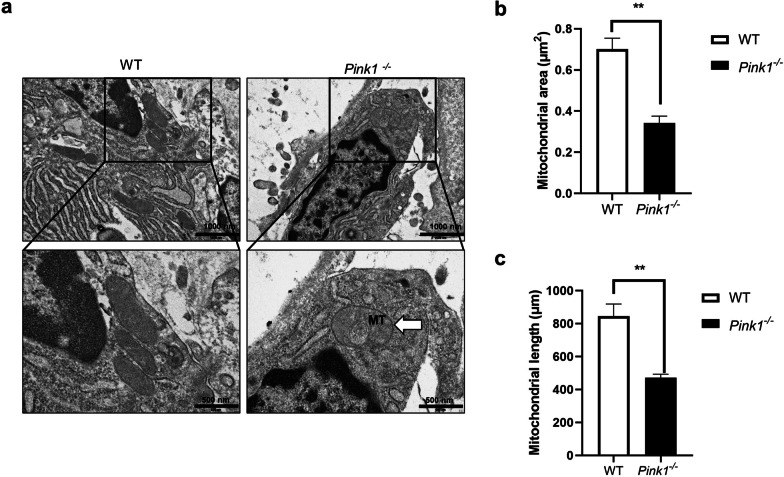
Morphology of mitochondria in osteoblasts and osteocytes of femurs from *Pink1*^−/−^ and WT mice. **a** Representative TEM of femurs from *Pink1*^−/−^ and WT mice. Boxed regions have been shown in an enlarged version. Mitophagy has been indicated using arrows. Upper image scale bar = 1000 nm, lower image scale bar = 500 nm (**b**–**c**). Quantitative analyses of morphometric data from TEM images have been depicted. TEM, Transmission electron microscope; MT, mitophagy. Data have been expressed as mean ± SEM; **P < 0.01; *n* = 3 per condition

We performed electron microscopy analysis on a subset of *Pink1*^−/−^ and WT mice femurs to evaluate their mitochondrial morphology. As shown in Fig. 5a, elongated mitochondria were observed in osteoblasts and osteocytes of WT mice, whereas short or spherically shaped mitochondria appeared in the cells of *Pink1*^−/−^ mice. Figure 5b shows decreased mitochondrial area in the osteoblasts of femurs from PINK1-deficient mice. In addition, quantitative analysis of transmission electron microscope (TEM) images indicated that there were more fragmented mitochondria in osteoblasts from *Pink1*^−/−^ mice, as compared to those from WT mice (Fig. 5c).

### PINK1 is downregulated in osteoporotic patients

**Fig. 6:**
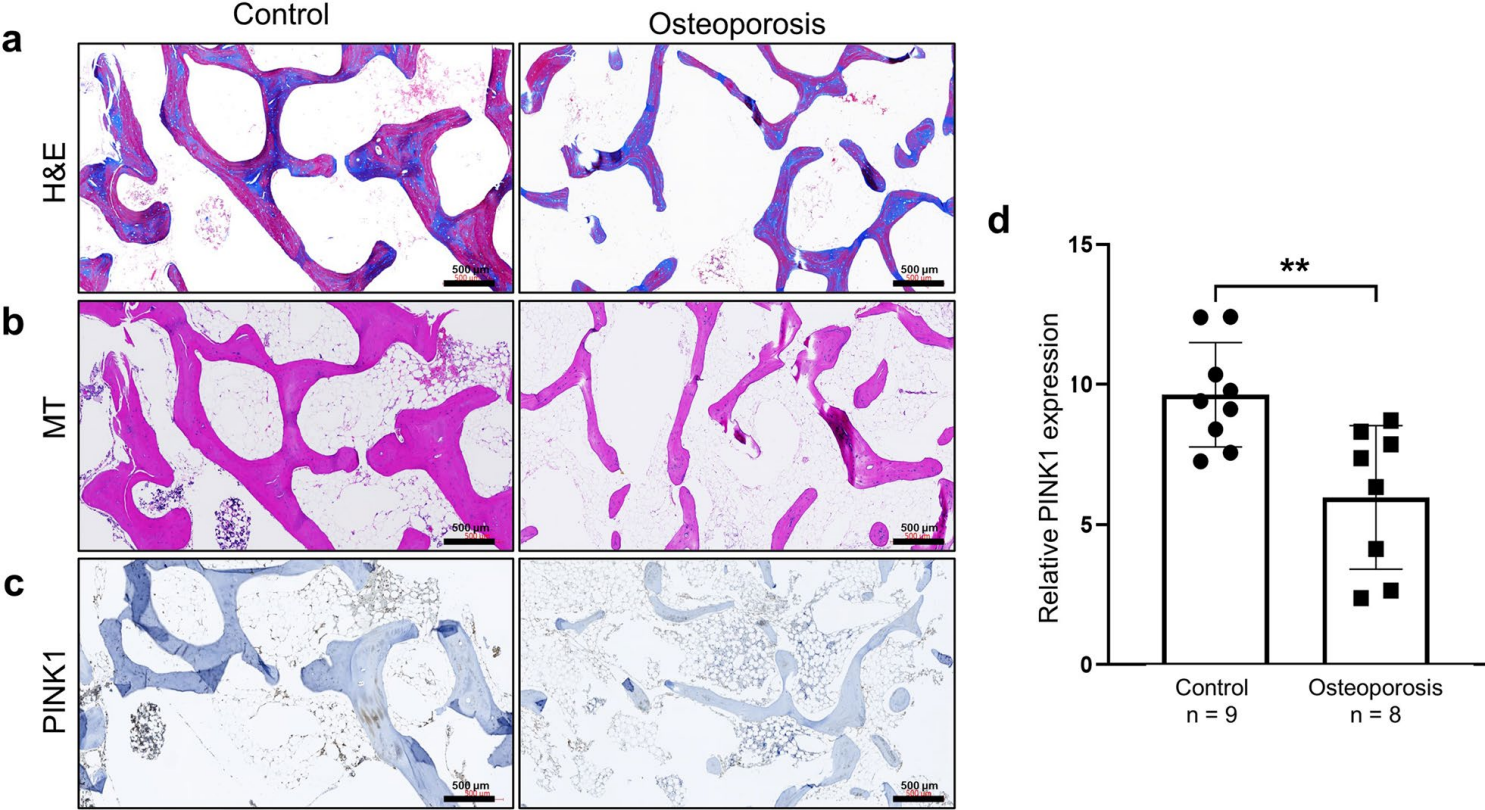
Downregulation of PINK1 expression in bones of patients with osteoporosis. Representative photographs of **a** hematoxylin–eosin (H&E), **b** Masson’s trichrome (MT) and **c** Immunohistochemical of PINK1 staining in femurs from patients with or without osteoporosis. **d** Comparison of PINK1 expression in femurs from patients with or without osteoporosis. Data have been expressed as mean ± SEM; **P < 0.01; scale bar = 500 μm

To confirm the clinical relevance of PINK1 as an osteoporotic marker, we identified the expression of PINK1 in bone tissues using frozen bone samples from patients with osteoporosis. Morphology studies using H&E revealed significantly lower Tb.N and Tb.Th in osteoporotic patients, than in control subjects (Fig. 6a). MT staining showed significantly decreased collagen fiber deposition (Fig. 6b). And, immunohistochemical staining showed lower expression of PINK1 in the bones of osteoporotic patients (Fig. 6c). We found that the expression of PINK1 was reduced in bone tissue homogenates from osteoporotic patients relative to those from control subjects (Fig. 6d).

## Discussion

In this study, we showed that expression of endogenous PINK1 increases during osteoblast differentiation and that genetic deletion of PINK1 aggravates the loss of bone mass in ovariectomized mice. PINK1 plays a role in mitochondrial biogenesis, networking, and mitophagy in osteoblast lineage cells. In addition, reduced PINK1 expression induced ROS overproduction and abnormal calcium uptake in the mitochondria of osteoblasts during differentiation. We confirmed the lower expression of PINK1 in bones of osteoporotic patients.

Mitophagy is an important way to maintain mitochondrial network homeostasis and protect cells from the deleterious effects of mitotoxicity [29]. The appropriate roles of mitochondria are considered vital for successful differentiation of osteoblasts [30, 31]. Undifferentiated cells have mitochondria with relatively low activity, and upon initiation of the differentiation process, there is a significant increase in mtDNA copy number, protein levels of respiratory enzymes, transcription of mitochondria-associated genes, oxygen consumption rate, and ATP content [30, 32] in osteoblasts. Defects in mitophagy could influence bone mass by failing to maintain healthy mitochondria during osteoblast maturation [33], but there are limited and inconsistent study results concerning mitophagy in bone diseases. Yang et al*.* [34] developed osteoporosis models in rats using dexamethasone and found that resveratrol, a Sirtuin-1 activator, shows protective effects on the loss of bone mass by promoting mitophagy in osteoblasts. In contrast, Zhao et al*.* [35] proposed that mitophagy negatively regulates osteogenesis, based on the observation of upregulated PINK1/Parkin pathways in type 2 diabetes-related osteoporosis.

We induced osteoporosis through OVX in mice, which is a commonly used model in studies with estrogen deficiency and postmenopausal processes in humans. Estrogen has protective effects on osteoblasts by stimulating osteoblast differentiation and function [36]. Therefore, estrogen deficiency allows clear manifestation of defects in osteoblasts. After OVX, *Pink1*^*−/−*^ mice exhibited further reduction in bone mass and lower collagen deposits with decreased Ocn, which is solely secreted by osteoblasts (Fig. 1). Consistent with the in vivo observations, *Pink1*-depleted osteoblasts showed impaired differentiation capability, including lower expression of osteoblast markers and decline in mineralizing matrix formation (Fig. 2). Our results suggest that upregulation of *Pink1* plays a crucial role in maturation of osteoblast.

*Pink1* is a core regulator of mitophagy, which is a form of selective macroautophagy of mitochondria [22]. PINK1 consists of an N-terminal mitochondrial targeting sequence (MTS), an α-helical transmembrane segment, and a serine/threonine kinase domain [33]. When PINK1 is localized to the surface of healthy mitochondria with normal Δ*Ψm*, the N-terminal MTS of PINK1 is transferred across the mitochondrial membrane [37], and PINK1 is cleaved twice by mitochondrial processing peptidase (MPP) and presenilin-associated rhomboid-like protease (PARL) at the mitochondrial matrix and inner membranes, respectively [38]. The cleaved PINK1 translocates from the mitochondria to the cytosol and rapidly undergoes degradation via the ubiquitin protease system [39]. Therefore, mitophagy is suppressed in normal mitochondria with a proton-gradient formed by mitochondrial respiration. When PINK1 is recruited at the surface of damaged mitochondria, PINK1 is not cleaved by MPP/PARL, due to depolarized ΔΨm, and accumulates on the mitochondrial outer membrane [12]. Activated PINK1 phosphorylates substrates, including Ubiquitin and Parkin, following recruitment of autophagy machinery, autophagosome formation, and eventual clearance of unhealthy mitochondria [13, 40–42]. In our study, suppression of PINK1 induced lower expression of autophagy-related proteins (LC3ii) and reduced lysosomes in an acidic environment, suggesting diminution of mitophagy (Fig. 3b and g). As a result, the proportion of depolarized mitochondria increased in osteoblasts after PINK1 suppression (Fig. 3d).

Interestingly, multifunctional roles of PINK1 beyond triggering mitophagy have been reported in mitochondrial quality control [12]. Mitochondria comprise of intracellular networks and undergo constant remodeling with fission and fusion which allows the mixing of the metabolites. This process depends on energy requirement or favorability of the environments. [43, 44]. PINK1 controls mitochondrial fission and fusion dynamics with or without Parkin, which enables selective removal of dysfunctional components [45]. Mitochondria-derived vesicles (MDVs) contain the outer membrane (or may include the inner membrane) and a matrix with a small diameter of 70–150 nm [46]. It has been demonstrated that PINK1 is involved in the generation of some MDVs and contributes to mitochondrial homeostasis, peroxisome biogenesis, and immune regulation [46–50]. In addition, PINK1 stimulates the generation of new mitochondria as replacement [12]. PINK1 directly phosphorylates PARIS; subsequently, PGC-1α restores its promoter activity and regulates the genes involved in energy metabolism associated with mitochondria [51]. A progressive loss of dopaminergic neurons in the substantia nigra has been found in mice after conditional knockdown of PINK1 linked to PARIS toxicity [51]. Furthermore, the roles of PINK1 in the translation of mitochondrial RNAs and import of the proteins have also been proposed in yeast and humans, for driving the local supply quickly [52–55]. In our current study, an increase in mitochondrial mass and DNA copy number was observed in osteoblasts after osteogenic induction, while attenuation in those increases was found in PINK1-deficient osteoblasts (Fig. 3c and e). After treatment with *Pink1* siRNA, upregulation of pro-fission proteins (Drp1 and Fis1), contrary to the downregulation of fusion protein (Mfn1), was observed in osteoblasts during differentiation (Fig. 3b). In addition, an increase in fragmented mitochondria was observed in osteoblasts and osteocytes of PINK1-KO mice (Fig. 5). This might be due to the failure of selective removal of unhealthy mitochondria due to PINK1 suppression, not by the direct effect of PINK1 on mitochondrial dynamics.

Although the radical forms of oxygen have harmful effects in cells, these have also been studied as regulators of cellular signaling [7]. Several lines of recent evidence strongly suggest that increased ROS levels or decreased antioxidant activities inhibit osteoblastogenesis [56]. Mouse models of premature aging present signs of oxidative damage and osteoporotic features [57, 58]. SOD2 (manganese-dependent superoxide dismutase)-KO mice show an increase in mitochondrial superoxide and significantly suppress osteoblast differentiation [6]. Estrogens and androgens are known to serve as protectors of osteoblasts by reducing oxidative stress in the extracellular-signal-regulated kinase (ERK)-dependent manner [59, 60]. ROS occurs primarily in the mitochondria due to the escape of electrons through the OXPHOS, which is responsible for the production of ATPs [61]. In our study, post *Pink1* siRNA treatment, there was an increase in the production of ROS in osteoblasts, indicating that mitochondrial quality control by PINK1 is a protective method against excess mitochondrial ROS development that allows further osteoblastogenesis to proceed normally.

Calcium ion is an important second messenger that participates in various cell signaling pathways [23]. Uptake of calcium ions by the mitochondria means energy production, mediated by the activation of mitochondrial enzymes, including ATP synthase [62]. Calcium ions in mitochondria are also involved in mitochondrial permeability transition pore opening and apoptotic cell death [63]. Using calvaria-derived osteoblasts, we showed that PINK1 deletion impaired mitochondrial transportation of calcium ions (Fig. 4). It is plausible that loss of PINK1 leads to the neglect of many dysfunctional mitochondria in osteoblasts, culminating in abnormal calcium uptake and reduction of energy production. It is noteworthy that PINK1 expression was downregulated in bones from patients with osteoporosis (Fig. 6), which supports the practical roles of PINK1 in human bone diseases.

Bone diseases, including osteoporosis and fractures, are related to morbidity, disability, and premature death [64]. Favorable progress of osteoblast differentiation and synthesis of bone matrix by the same are important to prevent and compensate for abnormal microstructural deterioration of the bone tissue. We demonstrated that there is increased expression of PINK1 in osteoblasts during differentiation, which is related to mitochondrial quality control and low ROS production. Hence, PINK1 deficiency resulted in low bone mass in our experiments. Enhancing PINK1 activity might be a possible treatment target in bone diseases, which can promote a healthy pool of functional mitochondria in osteoblasts.

## Materials and methods

### Mice and ovariectomized models

*Pink1*^*−/−*^ mice were generously gifted by Dr. Xiaoxi Zhuang (Department of Neurobiology, The University of Chicago). The mice were then backcrossed onto the C57BL/6 background for 20 generations. WT served as controls and were maintained under specific pathogen-free conditions at 23 ± 1 °C with 50% ± 10% humidity with a 12/12 h light/dark cycle. Food and water were provided ad libitum. Animal procedures were approved by the CHA University Animal Care and Use Committee (No. 200012). Methods were performed in accordance with approved guidelines, and all efforts were made to minimize the number of animals used and their suffering. Female mice were used for the OVX model experiments. The OVX models were generated by a 5-mm dorsal incision in 8-week-old female mice; a sham surgical control was also generated. The mice were randomly assigned into ovariectomized (*n* = 6) (OVX) or sham-operated (*n* = 6) (sham) groups in each WT and *Pink1*^−/−^ group. After 8 weeks, the OVX mice were sacrificed for further analysis.

### μCT analysis

μCT images of distal femurs fixed in 10% neutral-buffered formalin solution were achieved by using a high-resolution Skyscan 1173 Micro-CT system (Bruker, Aartselaar, Belgium). Images were acquired at an effective pixel size of 6.04 μm, voltage of 130 kV, current of 60 μA, and exposure time of 500 ms. Image reconstruction software (NRecon; Bruker) was used to reconstruct serial cross-sectional images with identical thresholds for all samples (0–6000 in Hounsfield units). Trabecular morphometry was evaluated by measuring BV/TV, Tb.N, Tb.Th, and Tb.Sp. 3D surface rendering images were created using a CT Analyzer (Bruker, Skyscan micro-CT) and Mimics version 20.0 (Materialize, NV, Leuven, Belgium).

A heatmap of Tb.Th was generated using smoothing, a wrapping function to obliterate unanalyzable particles. The processed model consisted of surface-only data; we had to re-mesh to create a 3D-volumetric mesh using Materialize-3-Matic 12.0 (Materialize, NV, Leuven, Belgium). Next, we created a heatmap of bone thickness using wall thickness analysis.

### Histological analysis

The mice and human femurs were decalcified using 10% ethylenediaminetetraacetic acid (EDTA; pH = 7.4) for 1 month. The EDTA solution was replaced every 2 days. The decalcified femurs were washed, dehydrated, and embedded in paraffin. Paraffin-embedded sections were prepared, dewaxed, and rehydrated. Five micrometer sections were cut and stained with standard H&E and MT. Immunohistochemistry of femurs was performed using a ready-to-use IHC/ICC kit (BioVision, Inc. CA), according to the manufacturer's protocol. Briefly, paraffin-embedded sections were deparaffinized, rehydrated, immersed in a retrieval solution (10 mmol/l citrate, pH 6.0), and then placed in a microwave for 10 min. Endogenous peroxidase activity was blocked using 3% hydrogen peroxide for 15 min. The slides were incubated in 3% H2O2 at room temperature for 30 min to quench endogenous peroxidase activity, and then blocked in blocking buffer (BioVision, Inc.) at room temperature for 15 min, followed by incubation with anti-PINK1 (ab23707; 1:100) at room temperature for 45 min. After incubation with HRP-anti-mouse or -rabbit IgG polymer at room temperature for 20 min and washing with PBS, the tissue sections were treated with 3,3'-diaminobenzidine at room temperature for 15 min, followed by counterstaining with hematoxylin at room temperature for 1 min.

### Transmission electron microscope analysis

Mice femurs were cut in to 5 mm cross sections, fixed in 1% osmium tetroxide for 1 h, and then decalcified. After washing with phosphate-buffered saline (PBS), the samples were dehydrated and embedded. Femur samples were cut into semi-thin (1 μm) and ultra-thin sections (80 nm) and stained with 1% uranyl acetate and lead citrate. The ultrastructure of cells and bone were analyzed using TEM (JEM-1230, Jeol, Japan).

### Cell culture and in vitro differentiation

The osteoblastic MC3T3-E1 cell line was cultured in α-minimum essential medium (α-MEM) supplemented with 10% (v/v) fetal bovine serum (FBS), 0.22% sodium bicarbonate, 100 U/mL penicillin, and 100 μg/mL streptomycin at 37 °C in a humidified atmosphere containing 95% air and 5% CO_2_. For in vitro differentiation, when the cell confluence was established, differentiation was initiated with 50 μg/mL ascorbic acid and 10 mM β-glycerophosphate in complete cell culture medium for the indicated periods, and the medium was exchanged every 2 days. After 7 days of osteogenic differentiation, cells were fixed in 4% paraformaldehyde for 10 min, and Alp staining was performed using NBT/BCIP staining kit, as stated in the manufacturer’s instructions. ALP activity was determined at 405 nm using reaction buffer and p-nitrophenyl phosphate as a substrate. ALP activity was calculated after normalization to total protein content. The protein content was measured using the bicinchoninic acid (BCA) method with a Pierce protein assay kit (Thermo Fisher Scientific, USA). Mineralized nodule formation was determined using ARS staining. Briefly, after osteogenic incubation for the indicated days, the cells were fixed in 95% ethanol for 20 min at room temperature, washed with distilled water, and finally stained with 0.1% ARS (pH = 4.2; Sigma-Aldrich. MO, USA) for 30 min. For quantitative assessment of the degree of mineralization, the stain was dissolved in cetylpyridinium chloride (Sigma-Aldrich, USA) and the absorbance at 570 nm was measured.

### Small interfering RNA and expression vectors

Small interfering (si)RNA duplex oligonucleotide targeting PINK1 (siPINK1; cat. no. 44599) and scrambled negative control siRNA (cat. no. 37007) were purchased from Santa Cruz Biotechnology, Inc. The expression vector for PINK1 was constructed using pcDNA3 plasmid (Invitrogen, MA, USA). The open reading frames (ORFs) of the PINK1 proteins were amplified by PCR and subcloned in frame into the pcDNA3 plasmid.

### Reverse transcription polymerase chain reaction

Total RNA was isolated from cultured cells using TRIzol® reagent (Thermo Fisher Scientific. MO, USA), as stated in the manufacturer’s protocol. To quantify mRNA expression, total RNA (1 μg) was reverse transcribed and standard reverse transcription was carried out using Transcriptase II (both from Invitrogen). Reverse transcription polymerase chain reaction was performed using PCR primers (Bioneer, Daejeon, Korea) and annealing temperatures listed in Table 1. 18S rRNA was used as an endogenous control. The signal intensity of the product was normalized to the respective 18S rRNA signal intensity.

**Table 1:** Osteoblast related marker primers used for RT-PCR

Gene name	Primer sequences (5'–3')	Annealing temperature (℃)	Product size (bp)
ALP	Forward: CCC ACG TTT TCA CAT TCG GT	57	190
	Reverse: GCC TGG TAG TTG TTG TGA GC		
BSP	Forward: TTT ATC CTC CTC TGA AAC GGT	55	110
	Reverse: GTT TGA AGT CTC CTC TTC CTC C		
OCN	Forward: GCG CTC TGT CTC TCT GAC CT	58	225
	Reverse: TTT GTA GGC GGT CTT CAA GC		
OPN	Forward: ACA CTT TCA CTC CAA TCG TCC	58	240
	Reverse: TGC CCT TTC CGT TGT TGT CC		
PINK1	Forward: CAC ACT GTT CCT CGT TAT GAA GA	56	157
	Reverse: CTT GAG ATC CCG ATG GGC AAT		
18S	Forward: CTG AGA AAC GGC TAC CAC ATC	58	107
	Reverse: GCC TCG AAA GAG TCC TGT ATT G		

*ALP* alkaline phosphatase, *BSP* bone sialoprotein, *OCN* osteocalcin; *OPN* osteopontin, *PINK1* PTEN-induced kinase 1

### Western blot

Cells were lysed by boiling in SDS sample buffer, resolved using SDS-PAGE, and transferred to nitrocellulose membranes. The membranes were blocked in 3–5% skim milk in TBST (10 mM Tris–HCl, pH 8.0, 150 mM NaCl, 0.05% Tween 20) at room temperature for 40 min and incubated with the specific primary antibody in the blocking solution at 4 °C overnight, then washed 3 times with TBST and incubated with horseradish peroxidase-conjugated secondary antibody. Finally, detection was done using an enhanced chemiluminescence system (Amersham Pharmacia Biotech, NJ, USA). Antibodies against ALP (sc-271431, 1:1000), OCN (sc-365797, 1:1000), OPN (sc-21742, 1:1000), BSP (sc-73630, 1:1000), PINK1 (sc-518052, 1:500), MFN1 (sc-166644, 1:1000), DRP1 (sc-271583, 1:1000), FIS1 (sc-376447, 1:1000), and β-actin (sc-47778, 1:3000) were purchased from Santa Cruz Biotechnology (TX. USA). Antibody against LC3ii (ab48394, 1:2000) was purchased from Abcam (MA. USA). Antibodies against Parkin and Tom20 (#4211, 1:1000), P62 (#42406, 1:1000) were purchased from Cell Signaling Technology (MA. USA). All the original blot images were provided in Additional file 1: Fig. S4.

### Real time RT-PCR for the assessment of mitochondrial DNA

Genomic DNA (gDNA) was isolated from the cultured cells using the Wizard® Genomic DNA Purification Kit (Promega, Madison, WI, USA) and quantified using a NanoDrop 2.0 spectrophotometer (Thermo Scientific, Foster City, CA, USA). To quantify the mtDNA/gDNA ratio, qPCR was performed to amplify one gene from the mitochondrial genome (human Nd1) and one gene from the nuclear genome (human β-globin). The primer was designed to include following sequences: Nd1 forward, 5′-CAA ACC GGG CCC CCT TCG AC-3′; Nd1 reverse, 5′-CGA ATG GGC CGG CTG CGT AT-3′; β-globin forward, 5′-GAG AAT GGG AAG CCG AAC ATA-3′; β-globin reverse, 5′-CCG TTC TTC AGC ATT TGG ATT T-3′.

### ΔΨm detection

To evaluate ΔΨm in the mitochondria of MC3T3-E1 cells, JC-1 dye was used according to the manufacturer's instructions (Mitochondrial Membrane Potential Assay Kit, #ab113850, Abcam, UK). JC-1 is a lipophilic, fluorescent cation that emit green fluorescence at low ΔΨm, while red fluorescence at high ΔΨm [65].

MC3T3-E1 cells were collected and stained with 10 µg/mL JC-1 at 37 °C in the dark for 15 min, followed by measurement of absorbance at the wavelengths of 590 nm (aggregate emission) and 530 nm (monomer species) in a microplate reader (Molecular Devices, Sunnyvale, CA, USA).

### Oxygen consumption rate (OCR)

Cellular oxygen consumption rate (OCR) of osteoblastic differentiating cells was measured using the Seahorse XF Cell Mito Stress Test kit (Agilent, Santa Clara, USA) and the Seahorse XFe96 Analyzer (Agilent) with triplicate. Control siRNA or PINK1 siRNA transfected differentiating MC3T3-E1 cells (at day 3) were seeded at 2 × 10^4^ cells/well on an XF24 plate at 24 h before the assay. On the day of the assay, the medium was changed to DMEM containing 584 mg/ml L-glutamine, glucose or bicarbonate, and the plates were incubated at 37 °C in an incubator without CO2 infusion for 1 h before running the OCR assay. The reagents for the assay were prepared using an XF Cell Mito Stress Test kit (Seahorse Bioscience, MA) according to the manufacturer’s protocol. Results were generated automatically using the Seahorse XF Mito Stress Test Reporter Generator.

### Mitochondria-specific fluorescence staining

The osteoblastic MC3T3-E1 cell line, maintained in Osteogenic Differentiation Medium (Cyagen Biosciences, CA, USA), was transfected with *Pink1* siRNA or control siRNA for 2 and 4 days. After washing, the cells were stained with MitoTracker™ Red (Beyotime, Jiangsu, China) solution for 30 min in the dark. Images were captured using an EVOS® FL Cell Imaging System (Thermo Fisher Scientific, MA, USA). For mitophagy assessment, cells were seeded on coverslips and transfected with *Pink1* siRNA or control siRNA for 48 h. Then, transfected with mtKeima-Red-Mito-7 (was purchased from Addgene, plasmid #56018) using the manufacture protocol of Lipofectamine 3000, following which the coverslips were examined using confocal microscopy (LSM-700; Carl Zeiss, Jena, Germany).

### Measurement of ROS

To evaluate intracellular and mitochondrial ROS production, the cells were incubated with 5 μM 2,7-dichlorofluorescein diacetate (DCF-DA; Molecular Probes) or 10 μM MitoSOX™ (Life Technologies. CA. USA) in osteogenic medium, 48 h after transfection with *Pink1* siRNA or control siRNA. The fluorescence intensity of the cells was captured using confocal microscopy (LSM-700; Carl Zeiss, Jena, Germany).

### Measurement of mitochondrial calcium levels

15-day-old WT and *Pink1*^*−/−*^ C57BL/6 mouse embryo calvarial cells were prepared by modifying the protocol of Liuz et al*.* [66]. Embryo calvarial cells were isolated from embryo calvaria, digested with 0.3% collagenase, and incubated in α-MEM plus with 100 U/mL penicillin and 100 μg/mL streptomycin and without FBS for 2 h in a shaking incubator maintained at 37 °C. After digestion, embryo calvaria were washed twice with PBS and filtered using a 100 μm strainer. Isolated cells were cultured at 37 °C in a humidified atmosphere of 95% air and 5% CO_2_. Cells were transfected with 200 ng of CMV-mito-R-GECO1.2 (a generous gift from Robert Campbell) plasmid in 96-well plates using Lipofectamine™ 2000 (Invitrogen. MA. USA). After 48 h of transfection, images of the mito-LAR-GECO1.2-expressing cells were taken using a confocal microscope (LSM-700; Carl Zeiss, Jena, Germany) equipped with a live cell chamber (XL S1, Karl Zeiss). Single-field confocal imaging in the region was conducted at time intervals of 10 s and continued for 90 frames (15 min). Fluorescence intensity was quantified as the average pixel intensity per cell using the same confocal imaging system.

### Patient specimens

Collection and use of osteoporosis patient specimens were approved by the Institutional Review Board of CHA University (CHA IRB#: 2018–05-036). Seventeen cases of specimens from nine of 62.4 ± 7.4-year-old female patient; normal (control) and eight of 61.8 ± 5.8-year-old female osteoporotic patients were obtained from the Department of Orthopedic Surgery, CHA Bundang Medical Center. The extracted specimens were immediately stored in liquid nitrogen. This study was conducted in agreement with the Declaration of Helsinki.

### Statistical analysis

All data have been expressed as mean ± SEMs of three independent experiments. All statistical analyses were performed using GraphPad Prism software 8 (GraphPad Software Inc. CA, USA). The comparison of two groups was done by using Student’s t test, while one-way ANOVA with Tukey's post hoc test was performed for the comparison of more than two groups. *P* < 0.05 was considered a statistically significant difference. Correlation between the expression levels of PINK1 was determined using Spearman’s analysis.

## Supplementary Information

**Additional file 1**. Representative Western blotting for the expression of PINK1 and internal control of β-actin in femur derived from 2-month-old *PINK1*^−/−^ or WT mice. Tissues were pooled from WT or KO mice (*n* = 3); experiments were repeated 3 times using mice from different litters.

## Data Availability

The authors confirm that the data supporting the findings of this study are available within the article and its supplementary materials.
