# Supplementary material for: PINK1 deficiency impairs osteoblast differentiation through aberrant mitochondrial homeostasis
Source: Stem Cell Res Ther. 2021 Nov 25;12:589. doi: 10.1186/s13287-021-02656-4 (PMC8614054; doi:10.1186/s13287-021-02656-4)

**Supplementary materials.**

**PINK1 Deficiency impairs osteoblast differentiation through aberrant mitochondrial homeostasis.**

**Short Title: PINK1 deficiency impairs osteoblast differentiation**

So-Young Lee^1^, Hyun-Ju An^1,2^, Jin Man Kim^3^, Min-Ji Sung^1^, Do Kyung Kim^4^, Hyung Kyung Kim^5^, Jongbeom Oh^2^, Hye Yun Jeong^1^, Yu Ho Lee^1^, Taeyoung Yang^1^, Jun Han Kim^2^, Ha Jeong Lim^1^, Soonchul Lee^2*^

^1^Department of Internal Medicine, CHA Bundang Medical Center, CHA University School of Medicine, Seongnam-si 13496, Republic of Korea

^2^Department of Orthopaedic Surgery, CHA Bundang Medical Center, CHA University School of Medicine, Seongnam-si 13496, Republic of Korea

^3^Department of Oral Microbiology and Immunology, School of Dentistry, Seoul National University, Seoul 03080, Republic of Korea

^4^CHA Graduate School of Medicine, 120 Hyeryong-ro, Pocheon 11160, Republic of Korea

^5^Department of Pathology, Kyung Hee University Hospital at Gangdong, Kyung Hee University, College of Medicine, Seoul 05278, Republic of Korea

S.-Y. Lee and H.-J. An contributed equally to the work as co-first authors.

* Corresponding Authors:

Dr. Soonchul Lee, Department of Orthopedic Surgery, CHA University School of Medicine, CHA Bundang Medical Center, 59 Yatap-ro, Bundang-gu, Seongnam-si, 13496, South Korea. Phone: +82-31-780-5289, Fax: +82-31-708-3578, E-mail: Lsceline78@gmail.com

**Supplementary Fig. S1.** The absence of PINK1 expression in mouse femur of *Pink1*^-/-^ mice was confirmed by Western blot.

**Supplementary Fig. S2.** The number of cells with osteocalcin positive expression were significantly lower in the *Pink1*^-/-^ mice than those in the WT. Data have been expressed as mean ± SEM; **P < 0.01.

**Supplementary Fig. S3.** The effects of PINK1 overexpression on the mRNA levels of marker genes of mature osteoblasts.

**Supplementary Fig. S4.** Western blot of cell lysate in figure. (a) Western blot bands of figure 2a (b) Western blot bands of figure 2b (c) Western blot bands of figure 3a (d) Western blot bands of figure 3b.

**Supplementary Fig. S1.**


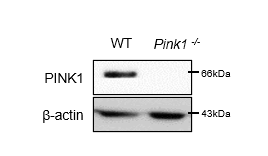


**Supplementary Fig. S2.**

WT Sham

WT OVX

*Pink1^-/-^* Sham

*Pink1^-/-^* OVX

**Supplementary Fig. S3.**


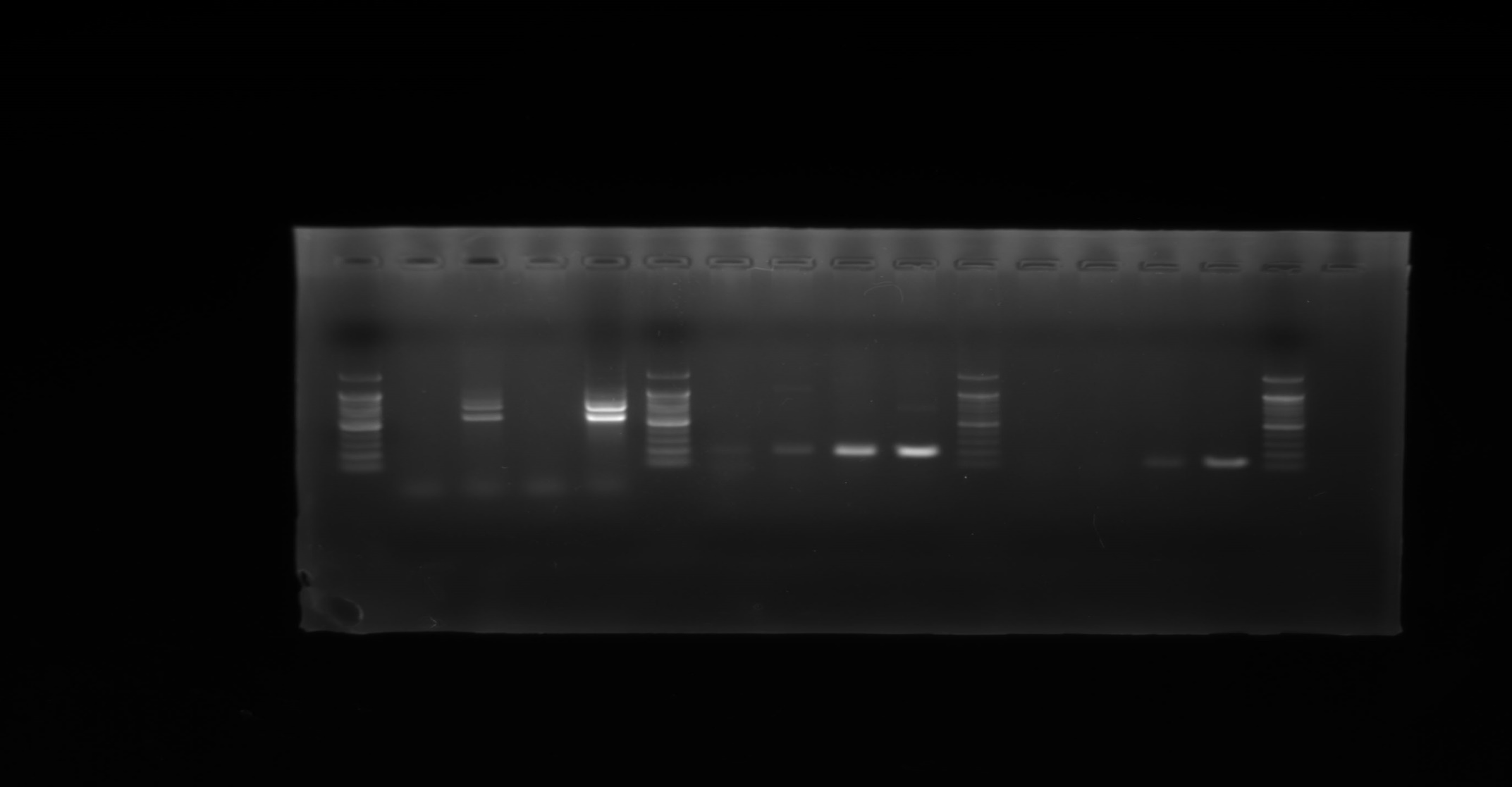

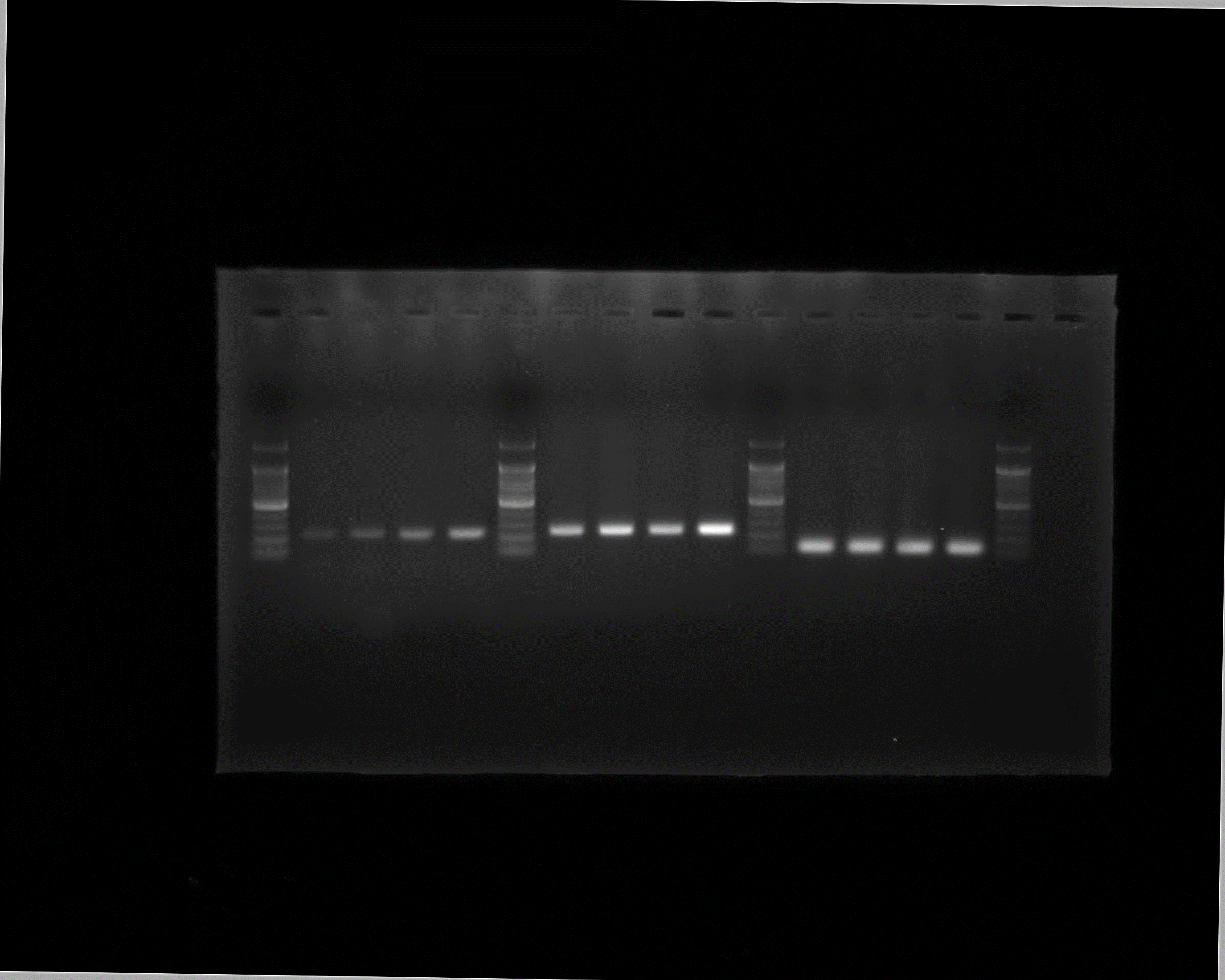

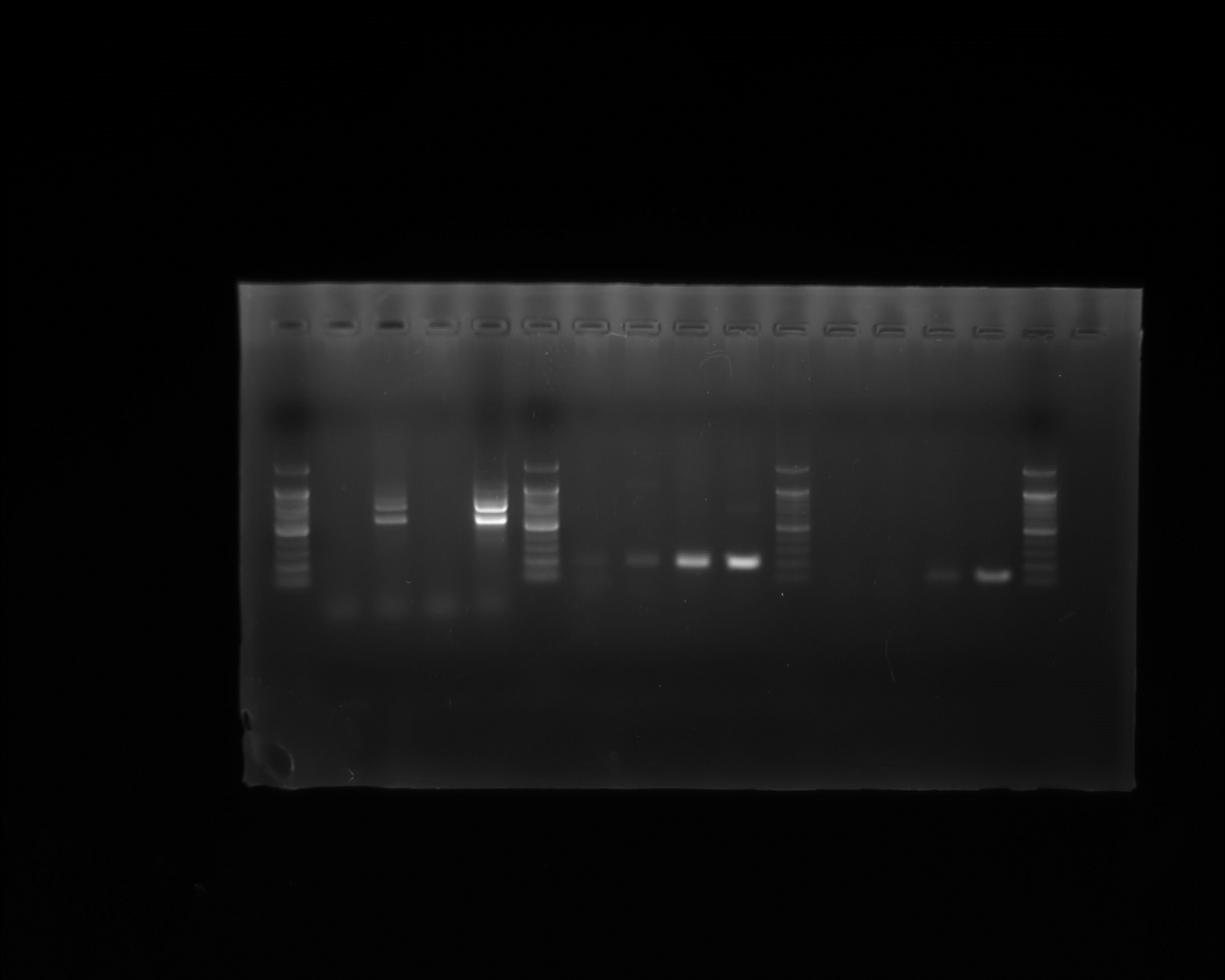

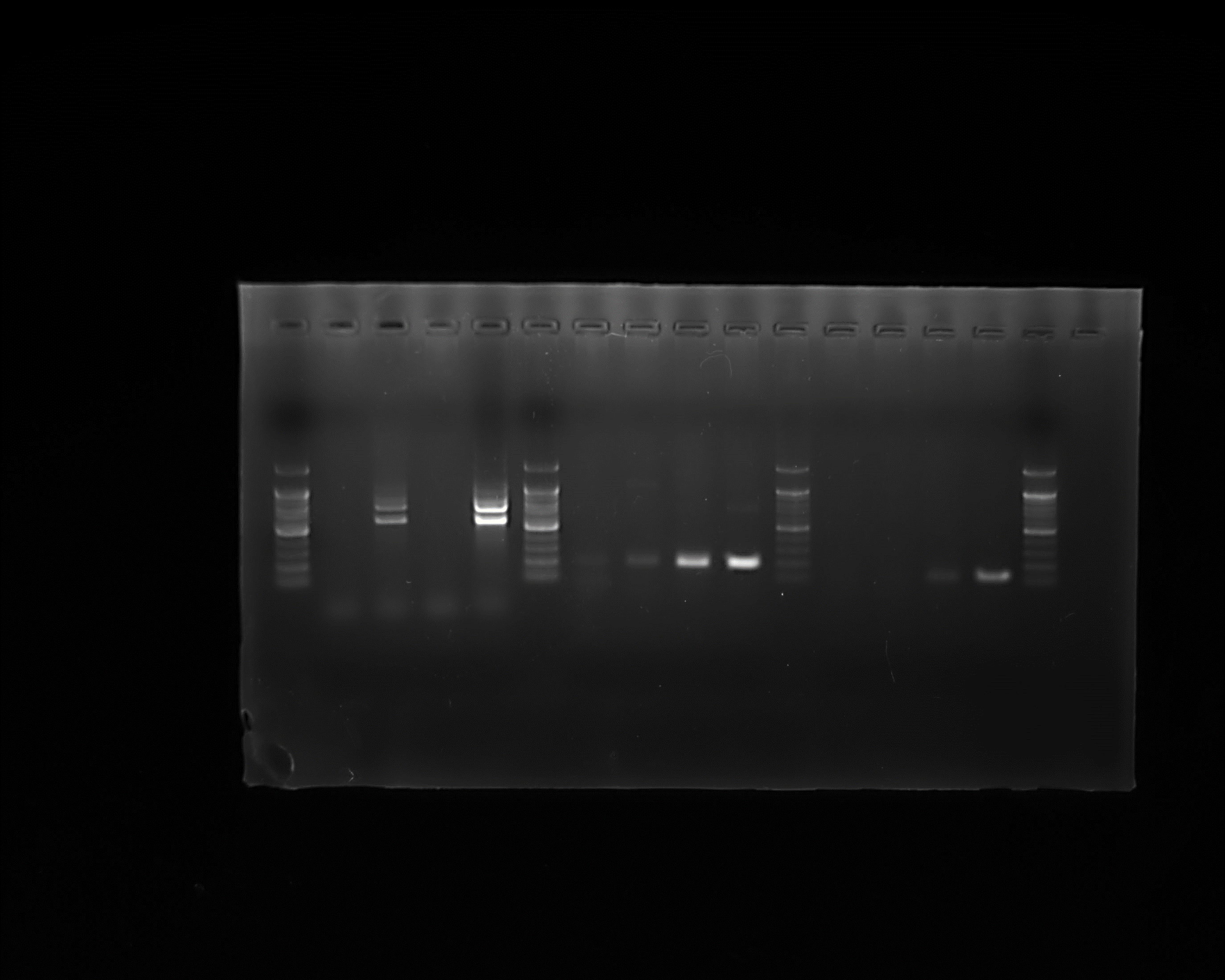

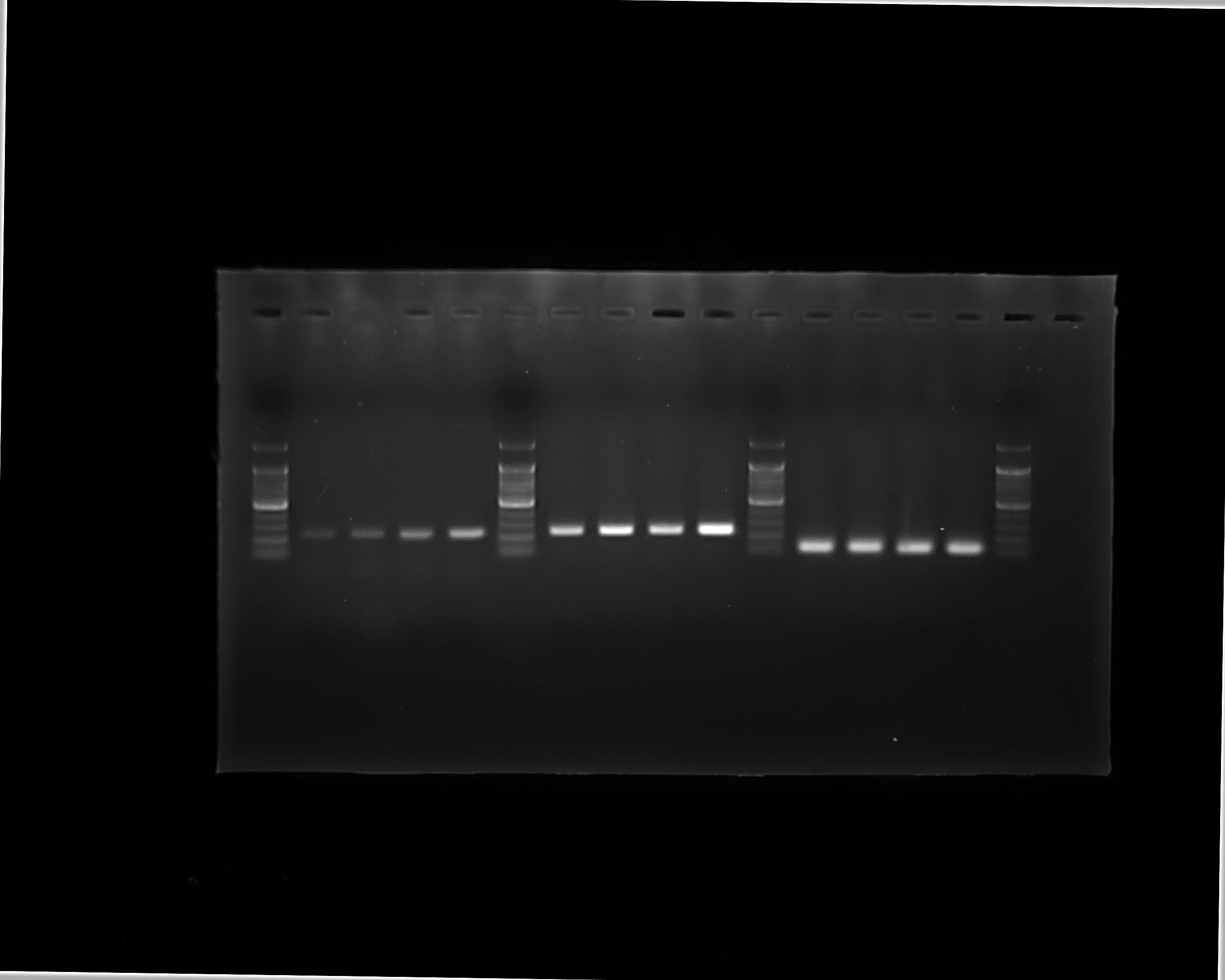

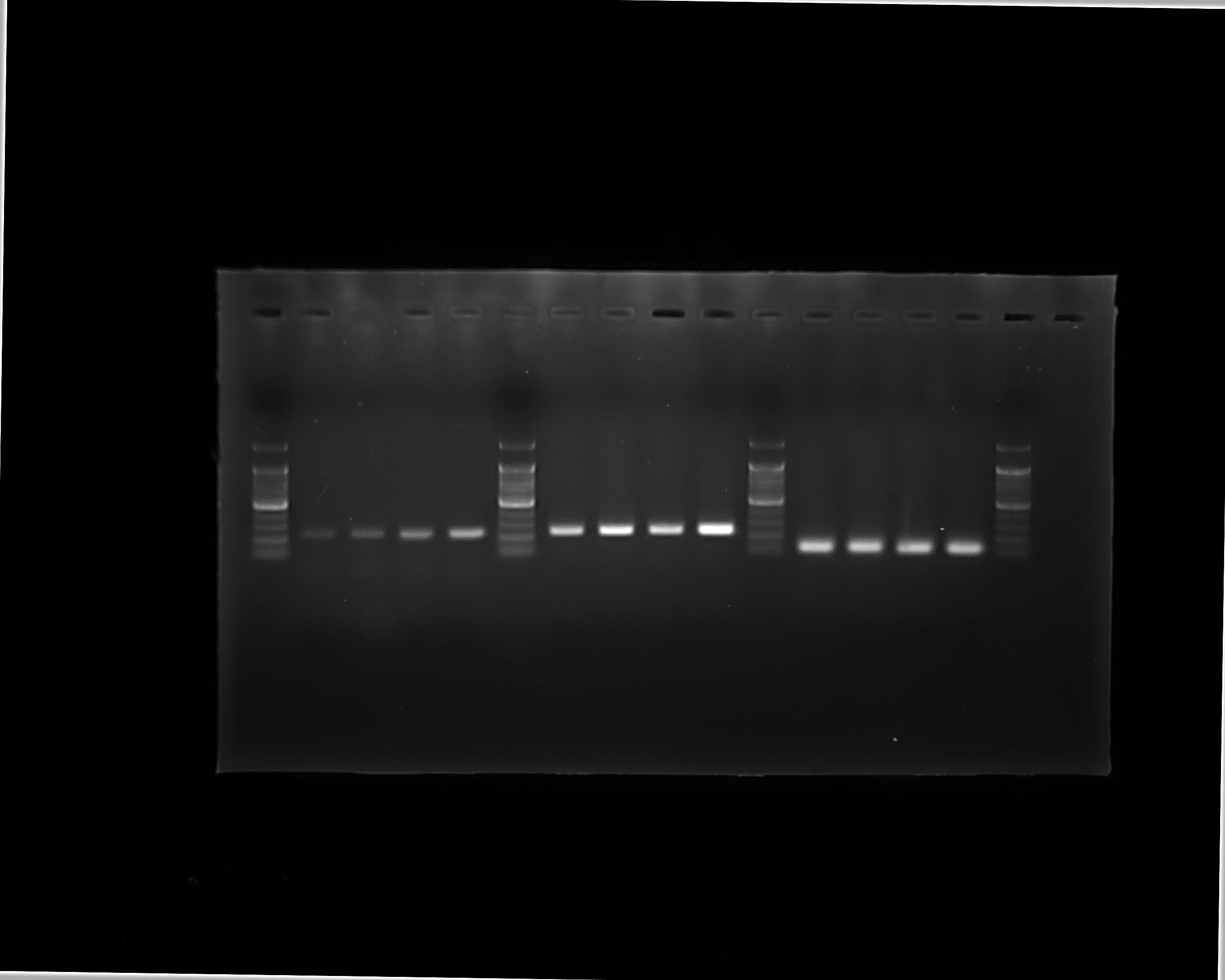


Alp

Bsp

Ocn

Opn

*Pink1*

18S

0

2

Days:

Differentiation

PINK1 O.E

Vector

PINK1 O.E

Vector

**Supplementary Fig. S4.**


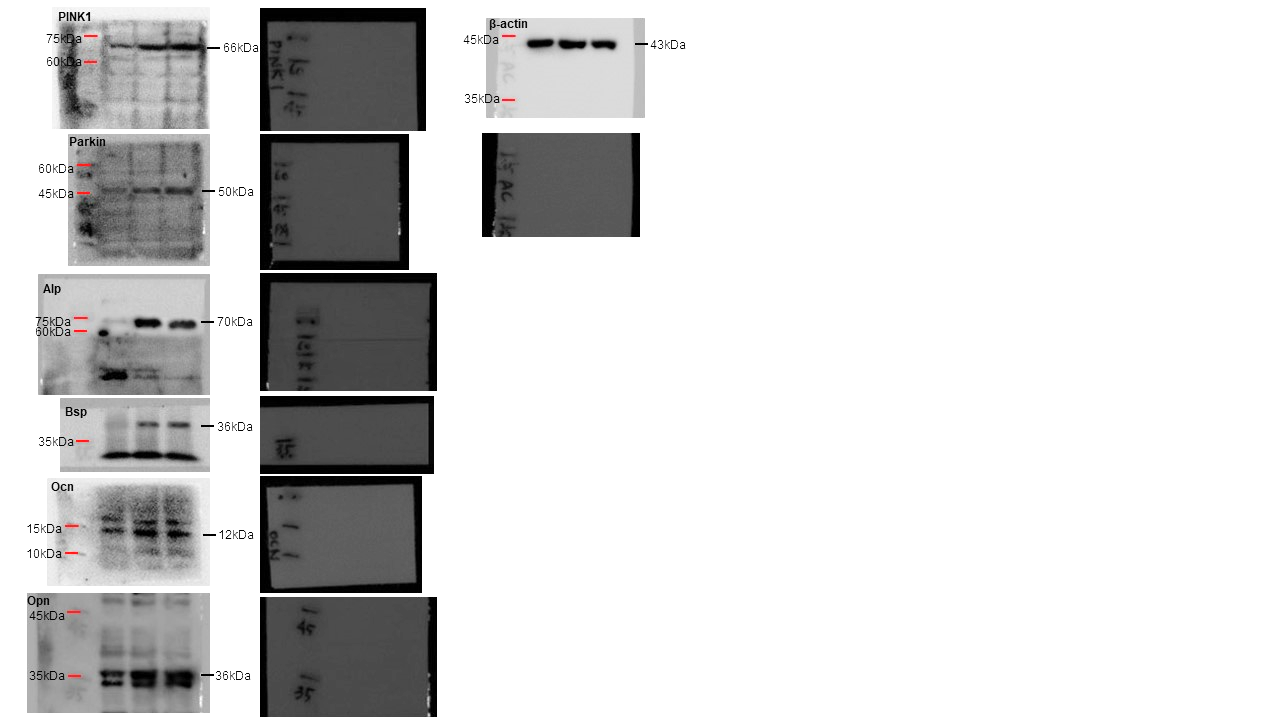
(a)

(b)


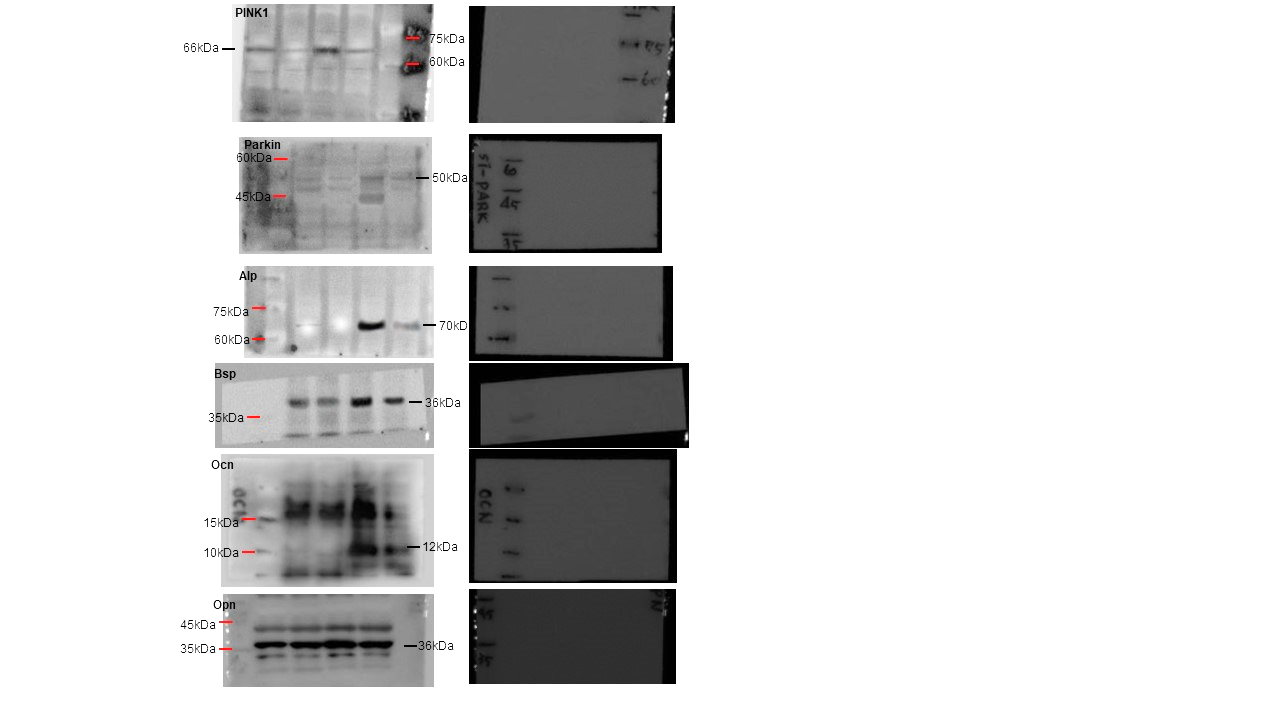


(c)


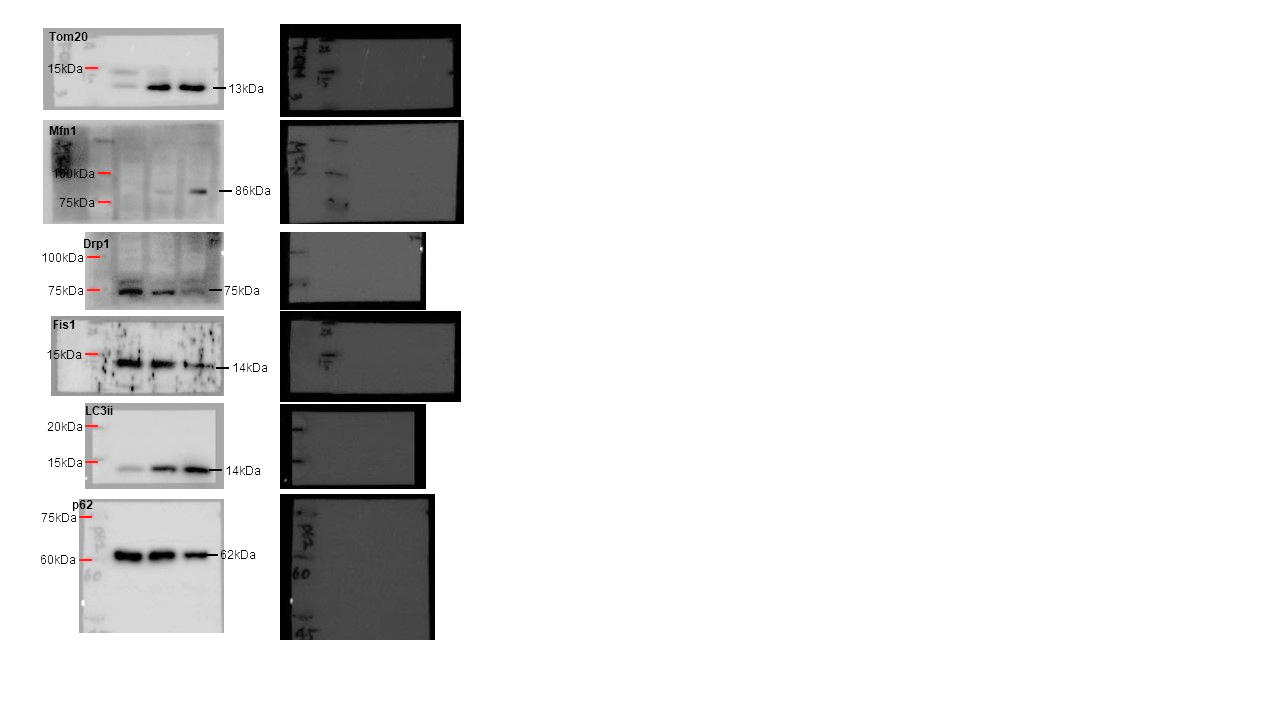


(d)
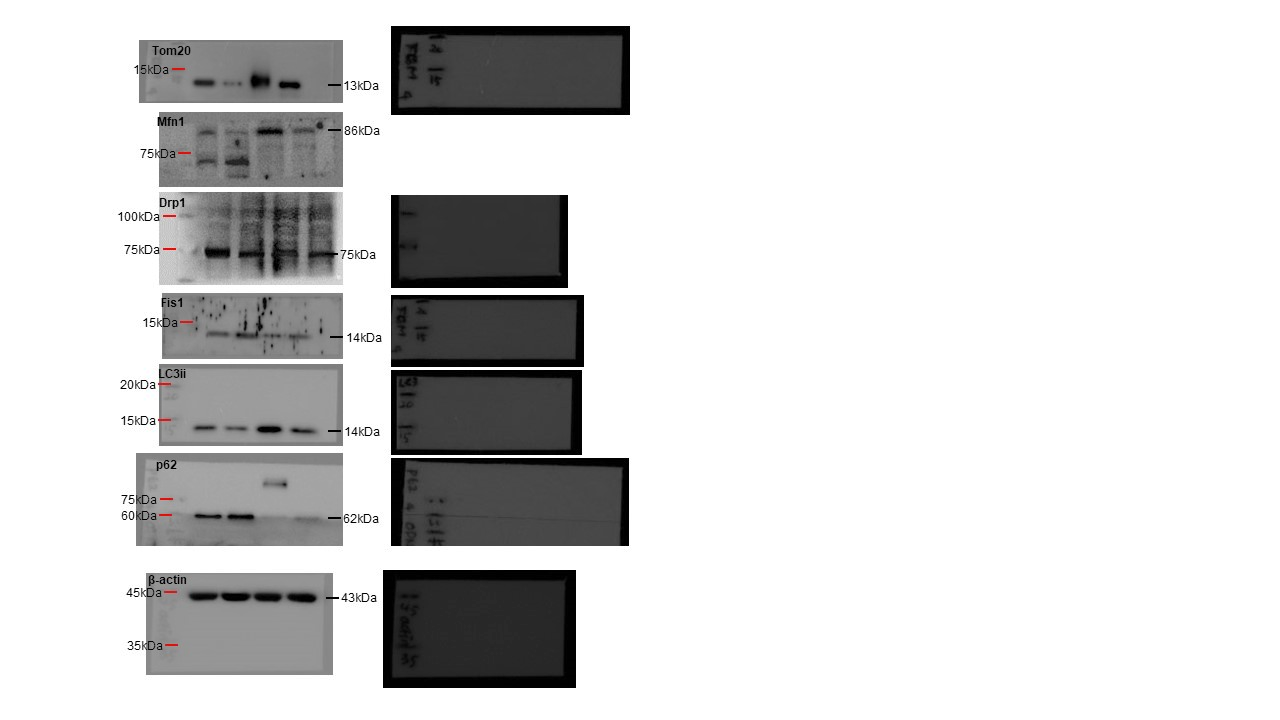

Supplement: Supplementary file 1 — Additional file 1. Representative Western blotting for the expression of PINK1 and internal control of β-actin in femur derived from 2-month-old PINK1−/− or WT mice. Tissues were pooled from WT or KO mice (n = 3); experiments were repeated 3 times using mice from different litters. [file 13287_2021_2656_MOESM1_ESM.docx]
